# Pregnancy and Cardiomyopathy After Anthracyclines in Childhood

**DOI:** 10.3389/fcvm.2018.00014

**Published:** 2018-03-26

**Authors:** Kara Annette Thompson

**Affiliations:** Department of Cardiology, University of Texas MD Anderson Cancer Center, Houston, TX, United States

**Keywords:** cardiotoxicity, pregnancy, anthracyclines, childhood, survivor

## Abstract

With advances in cancer therapy, there has been a remarkable increase in survival in children diagnosed with malignancies. Many of these children are treated with anthracyclines which are well known to cause cardiotoxicity. As more childhood cancer survivors reach childbearing age, many will choose to become pregnant. At this time, the factors associated with development of cardiomyopathy after anthracycline treatment are not clearly identified. It is possible that cardiac stress could predispose to cardiac deterioration in a patient with reduced functional reserve from prior anthracycline exposure. Pregnancy is one form of cardiovascular stress. The cardiac outcomes of pregnancy in childhood cancer survivors must be considered. In view of limited data, guidelines for pregnancy planning, management, and monitoring after cardiotoxic cancer therapy have not been established. This review summarizes the limited data available on the topic of pregnancy after anthracyclines in childhood.

## Introduction

Anthracyclines are effective antineoplastic agents that have contributed to the remarkable improvement in survival in children diagnosed with malignancy. Approximately 60% of childhood cancers are treated with anthracyclines. Unfortunately, these drugs are also well known to cause cardiotoxicity (1). The risk of cardiotoxicity is known to increase with higher cumulative doses (2). No dose is without cardiac risk (1). Yet, some patients can tolerate high cumulative doses (in excess of 1,000 mg/m^2^) without cardiotoxicity (3). Recently, topoisomerase (Top) 2B inhibition by anthracyclines has been identified as an important mediator of anthracycline-induced cardiotoxicity (4). Deletion of cardiomyocyte-specific Top2B has been shown to protect mice from anthracycline-induced cardiotoxicity, and patients with low levels of peripheral blood leukocyte Top2B have been shown to have higher resistance to anthracycline-induced cardiotoxicity. However, further study is needed before this information can be applied to clinical practice (5). As these drugs have high antineoplastic efficacy, and as it is not yet possible to identify patients with higher sensitivity, continued use of anthracyclines is expected despite the risk of cardiomyopathy with treatment. Thus, there will be an increase in the number of female childhood cancer survivors who reach childbearing age.

Cardiac dysfunction after treatment with anthracyclines is often subclinical. It has been reported that within 20 years, 25 to 50% of asymptomatic childhood cancer survivors have abnormal cardiac function found by echocardiographic evidence (1). The incidence increases with the length of time since treatment (6). Female sex and younger age at the time of cancer diagnosis are associated with increased risk of cardiac dysfunction (1). In young children, doxorubicin impairs myocardial growth resulting in a disproportionately small increase in left ventricular wall thickness in proportion to bodily growth. This excess afterload could contribute to the late development of myocardial deterioration (7). There is some evidence that anthracycline exposure results in reduced functional reserve such that additional cardiac stress could lead to acute deterioration (3). Ali et al. described 5 patients who developed a sudden decrease in fractional shortening in the setting of a history of a cumulative doxorubicin dose greater than 300 mg/m^2^ and development of viral infection (8). Pregnancy is another source of excess cardiac stress. The risk of development of cardiotoxicity with pregnancy in patients exposed to anthracyclines in childhood is not clearly known, but some limited data is available.

## Review of “Pregnancy After Anthracycline Literature”

In 1988, Davis and Brown published a case report of peripartum heart failure in a patient previously treated with doxorubicin. The patient was treated with 525 mg/m^2^ of doxorubicin at age six for osteosarcoma. At age 13, she developed acute systolic heart failure with an ejection fraction of 35% sixty-seven hours after delivering a full-term infant. She had an echocardiogram 2 years prior that showed normal systolic function. She underwent endomyocardial biopsy that showed cardiac fibrosis due to doxorubicin. She was thought to have subclinical doxorubicin fibrosis that lead to acute cardiac decompensation in the postpartum period (9).

In 1990, Goorin et al. reported 4 cases of initial congestive heart failure six to ten years after doxorubicin chemotherapy in childhood. One of the patients had development of symptoms 2 months after delivery of her first child and died 21 months later. This patient was noted to have reduced thickness of her left ventricular wall and increased afterload suggesting her cardiac development did not match her somatic growth (10).

In 1995, Lipshultz reported that female sex and higher rate of drug administration were independent risk factors for cardiac abnormalities after doxorubicin. Of the 12 patients with late congestive heart failure (mean of 10.2 years after doxorubicin treatment), 2 were women in the peripartum period (11).

In 1997, Katz et al. reported a case of a 28 year old primigravida patient who was diagnosed with acute heart failure 3 months postpartum. She had a history of large B cell lymphoma treated with 270 mg/m^2^ of doxorubicin ten years prior. Her ejection fraction 2 months after completion of therapy was 58% by radionuclide scintigraphy. Her ejection fraction 3 months postpartum was 20%. One year later, her ejection fraction remained severely decreased at 25% despite medical therapy (12).

In 2002, Pan and Moore reported the anesthetic management of 3 deliveries in 2 patients with doxorubicin-induced cardiomyopathy. The first patient was a 35 year old with a history of osteosarcoma and doxorubicin 20 years prior. She was active and asymptomatic through 28 weeks gestation and then developed shortness of breath and was found to have an ejection fraction of 30%. She further deteriorated and her ejection fraction decreased to 10%. She underwent an urgent cesarean section with combined spinal-epidural technique. The other patient had a history of doxorubicin treatment for Ewing’s sarcoma at age 16. She was active and had her first child at age 24 and developed heart failure with an ejection fraction of 35% five days after delivery. With medical therapy, her ejection fraction improved to 40–45%. She had a second pregnancy with a decrease in ejection fraction to 35%; vaginal delivery was successful with an epidural anesthetic. She had a third pregnancy and an ejection fraction of 25% at delivery. She had a combined spinal-epidural anesthetic and another successful vaginal delivery (13).

These case reports highlight the topic of doxorubicin associated cardiomyopathy with pregnancy. Asymptomatic female survivors of childhood cancer developed acute heart failure with pregnancy suggesting limited cardiac reserve after doxorubicin that was unmasked by the cardiovascular stress of pregnancy. Since these case reports, there have been 1 prospective and 3 retrospective reviews of this topic published, summarized in (Table 1).

**Table 1:** Comparison of studies of pregnancy in childhood cancer survivors (14–17).

	**Bar**	**Van Dalen**	**Hines**	**Thompson**
Type of study	Prospective	Retrospective	Retrospective	Retrospective
# of patients	37	53	847	58
Mean f/u time	17 years	20.3 years	26.5 years	20 years
Treatment/dose	<500 mg/m^2^ of anthracycline	267 mg/m^2^ of anthracycline	57% received anthracycline, 200 mg/m^2^	97% received anthracycline, 272 mg/m^2^
Definition of cardiotoxicity	FS <30% by echo	Signs and symptoms	Questionaires and echoes, SF <28%, EF <50%	EF <50% by echo
Conclusions	Pregnancy did not cause heart failure in those with normal baseline function; pregnancy did not increase risk of cardiotoxicity.	No heart failure occurred. Study was not powered to assess risk.	Pregnancy associated CMP in CCS was low but not insignificant, 1:500 vs. 1:3,000–4,000 in general population.	Subgroups identified with increased risk:1. younger age at time of cancer diagnosis2. Longer time from treatment to pregnancy3. Higher anthracycline dosePregnancy was an independent risk factor.

f/u, follow up; FS, fractional shortening; SF, shortening fraction; EF, ejection fraction; CMP, cardiomyopathy; CCS, childhood cancer survivor.

Bar et al. prospectively reviewed 37 women over 17 years who were followed in the same center for surveillance after childhood cancer and through pregnancy. These women were all treated with <500 mg/m^2^ of doxorubicin; only 4 received >450 mg/m^2^. Cardiac function was assessed by echocardiograms using measurement with fractional shortening (FS). FS <30% defined cardiac dysfunction. 29 women had FS ≥30% at baseline and had no change in cardiac function during pregnancy. 8 had FS <30% with a mean decrease of FS by 19% after pregnancy. This finding was not statistically significant, possibly due to the small number of patients, but suggested patients with baseline cardiac dysfunction might need special care. This study also suggested that pregnancy did not contribute to worsening cardiac function in those with normal baseline cardiac function and did not increase the risk of cardiotoxicity from prior anthracyclines (18).

Van Dalen et al. reviewed 53 childhood cancer survivors who delivered one or more children. Two of these patients had a history of acute congestive heart failure at the end of anthracycline therapy. In this study, the patients did not undergo routine evaluation with echocardiograms. Heart failure was defined based on clinical signs and symptoms. They received a mean cumulative dose of 267 mg/m^2^ of anthracycline therapy. None of the patients developed heart failure during pregnancy. Thus, it was not possible to evaluate risk factors for the development of heart failure, and the study was not powered to assess cardiac risk.

The largest review was published by Hines et al. in 2015 and included 847 female cancer survivors with 1,554 live births. Patients were identified by self-report from questionnaires sent to survivors treated at St. Jude Children’s Research Hospital between 1963 and 2006. Records were obtained to verify reports, and pregnancy-associated cardiomyopathy was defined as shortening fraction <28%, ejection fraction <50%, or treatment for cardiomyopathy during or up to 5 months after completion of pregnancy. Of the 847 patients, 484 (57%) received anthracyclines and 363 (43%) did not. The mean dose for those treated with anthracyclines was 200 mg/m^2^. Of the 847 survivors, 3 developed pregnancy-associated cardiomyopathy, 14 developed cardiomyopathy >5 months post-partum, and 26 were diagnosed with cardiomyopathy prior to pregnancy. This study suggests that the development of pregnancy-associated cardiomyopathy in childhood cancer survivors is low but not insignificant, with 3 in 1,514 births which is approximately 1 in 500. In comparison, peripartum cardiomyopathy occurs in the general population less commonly, in 1 in 3,000 to 4,000 live births. The risk for deterioration of cardiac function with pregnancy was worse in those with a known prior decrease in cardiac function; 8 of 26 patients or 30% decompensated in that group. This study was limited by possible underreporting as patients were identified by questionnaires, and it was not possible to calculate risk factors for development of pregnancy-associated cardiomyopathy due to the low numbers of patients with the outcome (14).

At MD Anderson, we evaluated the cardiac outcomes of childhood cancer survivors who had pregnancies and were previously exposed to anthracyclines and/or chest radiation. We identified 58 patients from the Children’s Cancer Hospital Longitudinal Database and the cardiology echocardiogram database who had pregnancies. We compared these women with pregnancies to a control group of 80 women from this same population with similar anthracycline dose and follow-up time who did not have pregnancies. 56 of the 58 women with pregnancies had received anthracyclines, and the mean dose was 272 mg/m^2^. Median follow up time was 20 years. Adverse cardiac events were defined as the presence or worsening of cardiomyopathy based on at least two echocardiograms showing an ejection fraction (EF) <50% or coronary artery disease (CAD). Peripartum was defined as during pregnancy or within 1 year after delivery. Of the 58 women who had pregnancies, 17 (29%) had an adverse cardiac event, 3 prior to pregnancy, 9 in the peripartum time frame, and 5 after pregnancy. 16 had decreased EF and 1 had CAD. Of the 17 patients with adverse cardiac events, 2 died (1 with CAD), 8 had recovered EF at last follow up, and 7 had decreased EF at last follow up. In comparison, of the 80 women in the control group without pregnancies, 12 (15%) had an adverse cardiac event. We identified subgroups with increased risk of adverse cardiac outcomes with pregnancy. Younger age at time of cancer diagnosis, longer time from cancer treatment to first pregnancy, and higher total anthracycline dose were associated with increased risk. Pregnancy was also identified as an independent risk factor with a 2.35 fold increase in cardiac risk (15).

The MD Anderson findings suggest more concern regarding cardiac outcomes in cancer survivors with pregnancies than the other studies. Bar et al. reported successful outcomes of pregnancy in women without significant LV dysfunction prior to pregnancy (18). In the MD Anderson data, follow up time was longer, and some patients who tolerated pregnancy developed cardiac dysfunction later. The two deaths in our group occurred 1 and 5 years after tolerating pregnancy. It is difficult to compare the MD Anderson study to the study by van Dalen et al. as the definition of cardiac dysfunction was based on echocardiogram findings rather than symptoms alone (16). In the study by Hines et al., only 0.3% of patients developed pregnancy-associated cardiomyopathy compared to 16% in the MD Anderson study. The Hines study was based on self-report rather than on echocardiographic findings. Thus, subclinical cardiomyopathy was likely under-reported (14). The MD Anderson study group was also a higher risk group; fifteen percent of survivors in the group who did not have a pregnancy had a cardiac event (15). Compared to the Hines group, the MD Anderson patients received a higher median dose of anthracycline. We had a higher proportion of patients who were treated for sarcoma with high doses of doxorubicin and fewer with acute lymphocytic leukemia who are usually treated with lower doses of anthracyclines. And, our overall population had a high mean cardiac risk score by the method of Chow (19).

## Discussion

**Figure 1:**
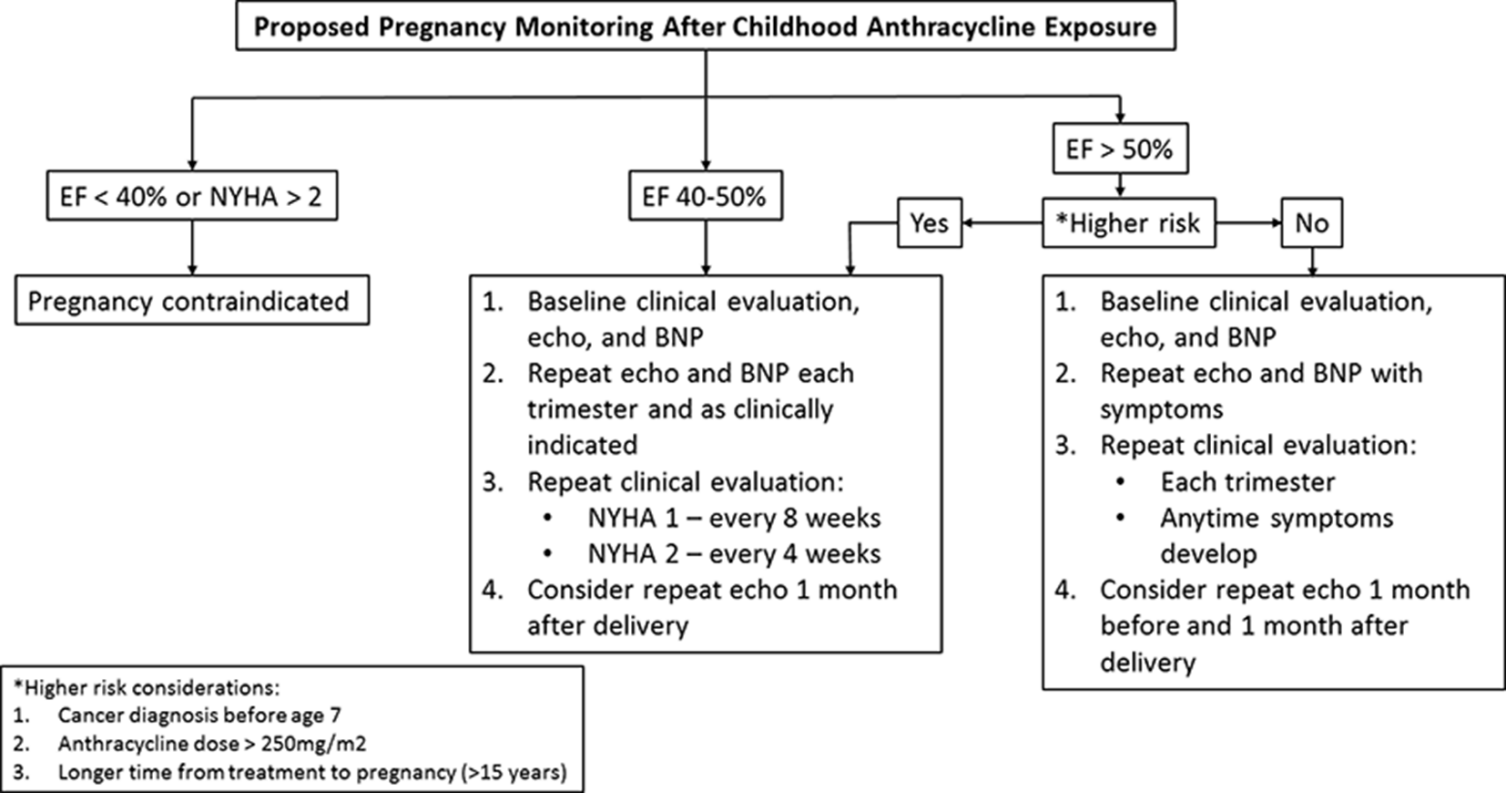
Proposed Monitoring Algorithm.

Pregnancy is associated with substantial changes in cardiac physiology and volume overloading. Yet, in normal hearts, chronic volume overloading with multiple pregnancies does not compromise left ventricular function (20). In patients with subclinical cardiac disease, the cardiac problem will often become manifest for the first time in pregnancy (21). In patients with a history of peri-partum cardiomyopathy, future pregnancies are generally not recommended, even if left ventricular (LV) function recovers as there can be an associated decrease in LV function and/or possible death. The risk seems higher in those who have a subsequent pregnancy with persistent LV dysfunction (22). Similarly, Siu et al. studied the outcomes of pregnancy in women with heart disease and identified four predictors of maternal cardiac complications and developed a risk index on the basis of these predictors. A prior cardiac event such as heart failure was one predictor which would be associated with a 27% estimated risk of a cardiac event with pregnancy according to this index (23). In patients with EF <40% or NYHA >2, pregnancy is not recommended (24). In patients with mild LV dysfunction or subclinical LV dysfunction, recommendations are less clear. For childhood cancer survivors, there are limited pregnancy guidelines based on minimal data. International guidelines suggest that surveillance for cardiotoxicity is reasonable before pregnancy or in the first trimester for all women previously treated with anthracyclines and/or chest radiation (25). There are no recommendations regarding ongoing surveillance in pregnant cancer survivors who have normal left ventricular systolic function prior to pregnancy or in the first trimester (2). Dutch guidelines recommend that all pregnant cancer survivors treated with any cardiotoxic treatment should have an echocardiogram in the third trimester of pregnancy (26). With the increasing number of childhood cancer survivors reaching child-bearing age, it would be ideal to develop pregnancy monitoring guidelines for this group of patients. For the present time, it is important for patients and doctors to recognize that there is increased cardiac risk with pregnancy in patients previously exposed to anthracyclines. Higher risk factors to consider based on MD Anderson data include younger age at time of cancer diagnosis, higher anthracycline dose, and longer time from cancer treatment to pregnancy. In addition, pregnancy is possibly an increased risk factor for development of cardiomyopathy in patients previously exposed to treatment with anthracyclines and/or radiation (15). Until registries are established to collect data and develop formal guidelines, I propose the following monitoring algorithm (Figure 1).

## Author Contributions

The author confirms being the sole contributor of this work and approved it for publication.

## Conflict of Interest Statement

The author declares that the research was conducted in the absence of any commercial or financial relationships that could be construed as a potential conflict of interest.
